# The Use of High-Flow Nasal Cannula and the Timing of Safe Feeding in Children with Bronchiolitis

**DOI:** 10.7759/cureus.15665

**Published:** 2021-06-15

**Authors:** Thomas P Conway, Claudia Halaby, Meredith Akerman, Arsenia Asuncion

**Affiliations:** 1 Pediatric Emergency Medicine, Northwell Health, Queens, USA; 2 Pediatric Pulmonology, NYU Winthrop, Mineola, USA; 3 Biostatistics, NYU Winthrop, Mineola, USA; 4 Pediatric Critical Care, NYU Winthrop, Mineola, USA

**Keywords:** bronchiolitis, high flow, feeding, oxygen, infant

## Abstract

Objective

The use of high-flow nasal cannula (HFNC) as non-invasive respiratory support in children with bronchiolitis has increased over the last several years. Several studies have investigated enteral feeding safety while on HFNC. This study compares the safety of oral feeding prior to and following implementation of an HFNC feeding guideline.

Patients and methods

A retrospective study was designed, in children ≤2 years of age with bronchiolitis, requiring HFNC, from 2017 to 2019. We defined feeding complications on HFNC and defined safety as the absence of such complications. We gathered the following data: oral feeding timing from the HFNC initiation, duration of enteral feeding on HFNC, and HFNC flow rate at which the feeding was initiated. We compare the data prior to and post-implementation of an HFNC feeding guideline.

Results

Descriptive statistics were calculated separately by pre and post guideline implementation. Patients in both pre and post guideline implementation groups had no feeding complications on HFNC. Subjects in the post (n=50) vs. pre-guideline implementation (n=36) had a higher median amount of liters flow when initiating enteral feeding (8.0 vs. 6.0 respectively, p<0.024), spent fewer days in the pediatric intensive care unit (PICU) (two days vs. 0 days). Post guideline implementation, enteral feeding was initiated sooner (days *nil per os *[NPO] 1.0 vs 2.0). No other significant differences between the two cohorts with respect to other variables were observed.

Conclusions

Our data supports that oral feeding in patients with bronchiolitis on HFNC is safe. Utilization of current guidelines allowed safe earlier feeding of children on HFNC, reducing the time spent NPO.

## Introduction

Viral bronchiolitis is a major cause of pediatric hospitalization. There are over 100,000 bronchiolitis admissions in the United States annually, requiring supportive care and, less frequently, supportive oxygen therapy [1]. In concordance with the 2014 bronchiolitis guidelines from the American Academy of Pediatrics (AAP), physicians are recommended to avoid treating these patients with inhaled beta-agonists, systemic corticosteroids, antibiotics, and even continuous pulse oximetry [2]. Nevertheless, infants with bronchiolitis remain a high-risk population with frequent hospitalizations for both hypoxemia and dehydration.

Although hypoxemia had been treated with traditional nasal cannula oxygenation in the past, the advent of high-flow, humidified, warmed oxygen has become a new tool in the inpatient management of bronchiolitis. A noninvasive form of respiratory support, high-flow nasal cannula (HFNC) provides several benefits including a reduction in energy expenditure, nasopharyngeal hypercarbic washout, and generation of a degree of airway distending pressure, increasing functional residual capacity (FRC) of the lungs. The advantage of HFNC allows for the potential of early oral feeding, which has been shown to decrease the length of stay in critically ill pediatric patients [3]. Controversy remains on when to initiate oral feeding while on HFNC and the safety and efficacy surrounding its use. Convention wisdom has cautioned against the initiation of feeding on infant respiratory support due to the risk of aspiration. 

In implementing an HFNC guideline, we aimed to standardize practice, particularly with regards to starting oral feeding safely. The primary outcome of this study was to measure the presence of feeding complications of patients while on HFNC after the implementation of the guideline. The secondary outcomes were the timing of initiation of oral feedings and the measurement of average liters flow on which feeding was initiated.

## Materials and methods

Study design

This study was a retrospective chart review performed in a University Affiliated Pediatric Program from September 1, 2017, until May 31, 2019, investigating the use and safety of HFNC and oral feeding. The subjects included children under two years old admitted to a pediatric inpatient ward or the intensive care unit with a primary diagnosis of bronchiolitis. The study was approved by the hospital institutional review board (IRB).

**Figure 1:**
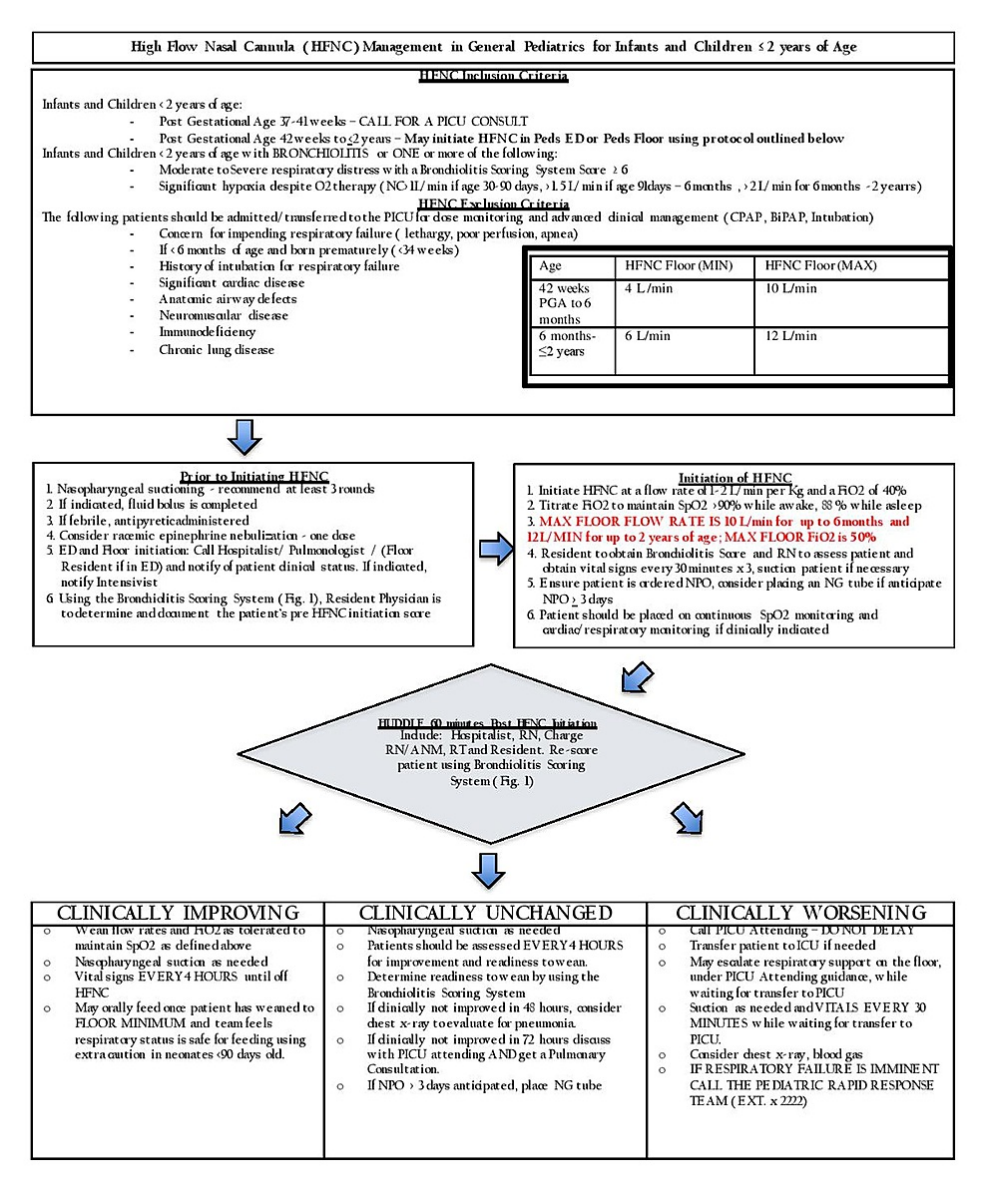
High-flow nasal cannula management guideline for bronchiolitis in the pediatric ward HFNC: high-flow nasal cannula; PICU: pediatric intensive care unit; ED: emergency department; NC: nasal cannula; CPAP: continuous positive airway pressure; BiPAP: bilevel positive airway pressure; PGA: post-gestational age; FiO2: fraction of inspired oxygen; SpO2: oxygen saturation; RN: resident nurse; NPO: *nil per os*; NG: nasogastric; ANM: auxiliary nurse midwife; RT: respiratory therapist

**Figure 2:**
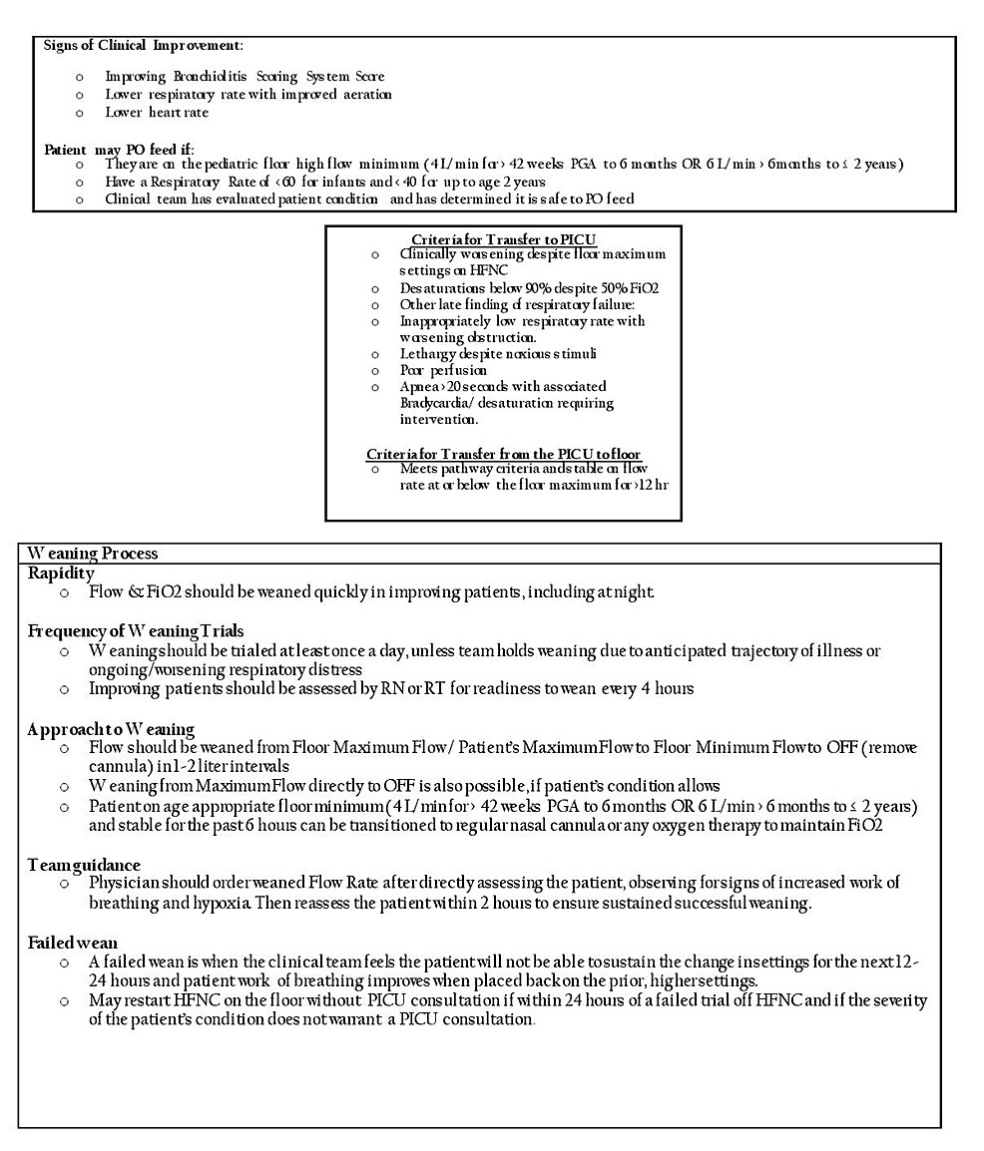
High-flow nasal cannula management guideline for bronchiolitis in the pediatric ward PO: *per os*; PGA: post-gestational age; PICU: pediatric intensive care unit; HFNC: high-flow nasal cannula; FiO2: fraction of inspired oxygen; RN: resident nurse; RT: respiratory therapist

**Figure 3:**
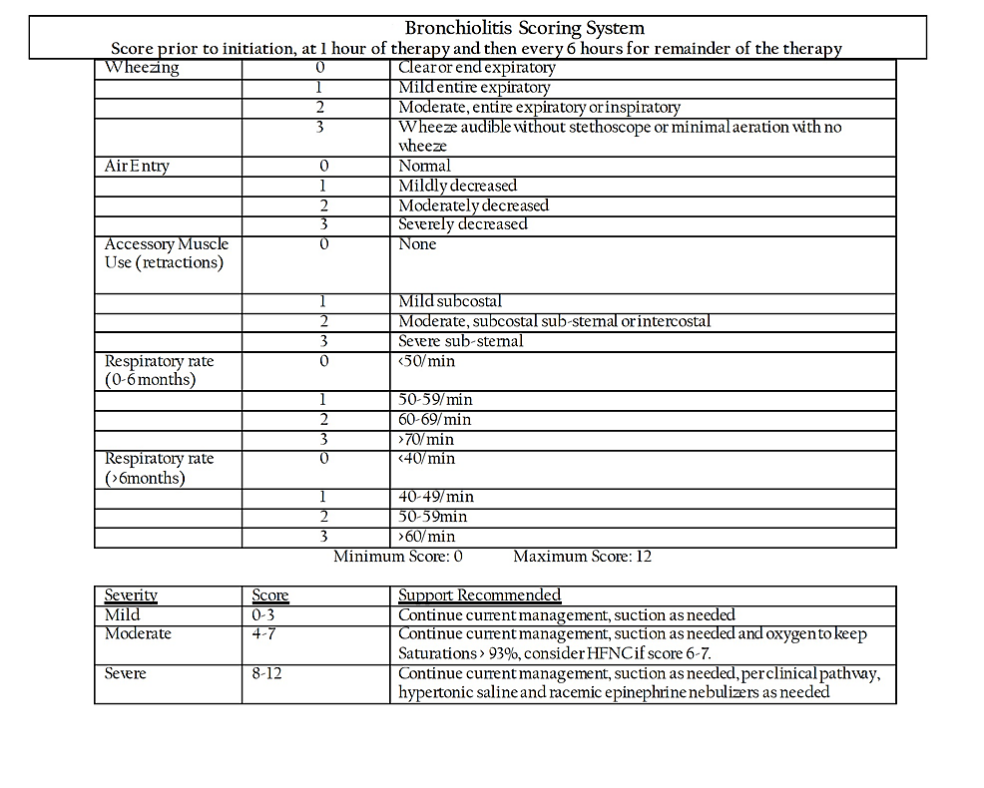
High-flow nasal cannula management guideline for bronchiolitis in the pediatric ward HFNC: high-flow nasal cannula

In the fall and winter of 2017, due to the increasing needs of bronchiolitis patients requiring HFNC and the limited number of intensive care unit (ICU) beds, HFNC was started in the inpatient unit. These patients required pulmonary and critical care consults, and no inpatient guidelines or decision-making tool for the initiation and titration of HFNC existed. In 2018, HFNC inpatient and pediatric ICU guidelines were created. After guideline initiation, these patients were managed in the general ward under the pediatric hospitalist service (Figures 1-3) [4]. In addition, these guidelines included criteria on the initiation of patients' oral feeding regimens. All post-guideline patients strictly adhered to the recommendations set forth as noted in documentation obtained from nursing, respiratory care, and resident physician electronic medical documentation. Therefore, after the introduction of a formalized process for using an HFNC, we analyzed the data in order to recognize if a formal guideline changed the management and comfort of practitioners feeding these patients by mouth. This retrospective study analyzed patients' data to compare whether there is a difference in the pre-guideline and post-guideline phases in terms of timing of initiating oral feeding and possible feeding complications associated with the use of HFNC.

Data collection

The study subjects were identified from the electronic medical record (EMR) based on the inclusion criteria of the age of <2 years and ICD-10 code for acute bronchiolitis (J21.8) clinically. The EMR was then queried for the following data: respiratory support, length of stay, inpatient or ICU admission, day of initiation of both nasogastric and oral feeding, and the amount of oxygen and flow rate (liter/kg) utilized during the initiation of oral feeding. 

The following data were also collected by query of individual chart review, searched by medical record number and obtained through daily progress notes: subject’s demographics such as age and gender, days spent without enteral feeding, days using a nasogastric tube for feeding, number of pediatric ICU or inpatient days, number of HFNC days, and HFNC liter/kg when enteral feeding was initiated. The subject’s weight was recorded and a mean liter per kilogram ratio was calculated at the start of the first oral feeding. HFNC oxygenation was defined as heated and humidified oxygen delivered at a flow rate greater than 4 L/min through a standardized HFNC (Fisher and Paykel) apparatus. The initiation of oral feeding as well as the log of adverse events was extracted from a daily pediatric resident, nursing, and respiratory therapist’s progress notes. Adverse events were defined as increased work of breathing within one hour at the start of feeding, vomiting, and increased HFNC settings. Safety of enteral feeding was defined as the absence of the above feeding complications.

Inclusion and exclusion criteria

All children under two years old with a clinical diagnosis of bronchiolitis who required HFNC were included in the study. Data were separated and compared between the two groups both before and after the initiation of HFNC guidelines. We excluded from our study children with congenital heart disease, cystic fibrosis, immunosuppression, or neuromuscular disease.

Statistical analysis

Descriptive statistics were calculated separately by pre and post guideline implementation. Comparison of the two groups was done using chi-square test for categorical variables and Mann-Whitney for continuous data, and statistical significance was at p<0.05 level. Analyses were performed using SAS version 9.4 (SAS Institute Inc. Cary, USA). 

## Results

From September 2017 until May 2019, 87 children were hospitalized with the diagnosis of bronchiolitis and requiring HFNC for respiratory support and fed by oral route. Only one child on short-term HFNC was excluded due to pre-existing cardiac disease, requiring intubation almost immediately upon admission. Patients included in the study were admitted to either the ICU or general floor. There was a total of 36 subjects on HFNC admitted prior to the implementation of the current feeding on HFNC guideline and 50 subjects on HFNC post guidelines, with one subject excluded due to cardiac disease. No statistical differences were recorded between the study groups in terms of age, hospital length of stay, or weight (Table 1).

**Table 1:** Demographics of pre-guideline vs. post-guideline patients fed on HFNC respiratory support HFNC: high-flow nasal cannula; NS: not significant

Variables	Pre-guideline (n=36)	Post-guideline (n=50)	p-value	p<0.05
Age (months)	4.5 (2.5, 8.0)	6.0 (3.0, 17.0)	0.124	NS
Length of Stay (days)	7.0 (5.0, 9.0)	6.5 (5.0, 9.5)	0.676	NS
Weight (kilograms)	8.2 (5.0, 10.3)	7.3 (5.1, 11.0)	0.990	NS

Our study showed that with the utilization of HFNC guidelines (Table 2), the majority of the patients could be managed safely in the inpatient service and oral feeding could be initiated at a higher HFNC rate/kg with no adverse events. Although days of *nil per os *(NPO) showed no statistical significance (2.0 vs 1.0 days ) between the two groups, the data showed that children with bronchiolitis started oral feeding earlier in the post-guideline group.

**Table 2:** Pre guideline implementation vs. post guideline implementation of children on HFNC respiratory support *** Significant; NS: not significant; N/A: not applicable NPO: *nil per os*; PO: *per os*; PICU: pediatric intensive care unit; HFNC: high-flow nasal cannula; NG: nasogastric

Variables	Pre-guideline (n=36)	Post-guideline (n=50)	p-value	p<0.05
Days NPO	2.0 (0.0, 2.0)	1.0 (0.0, 1.0)	0.103	NS
NG Feeding (days)	0.0 (0.0, 3.0)	0.0 (0.0, 1.0)	0.229	NS
PICU Admissions	24 (66.7%)	19 (38.0%)	0.0087	***
Floor Admissions	12 (33.3%)	31 (62.0%)	0.0087	***
Mean PICU days	2.0 (0.0, 5.0)	0.0 (0.0, 2.0)	0.004	***
Days in HFNC	3.0 (2.0, 4.0)	3.5 (2.0, 5.0)	0.020	***
High Flow Liters on which PO started (L)	6.0 (4.0, 8.0)	8.0 (6.0, 10.0)	0.005	***
Adverse Events	0	0	N/A	

Table 2 highlights the statistically significant decrease in the amount of time spent admitted in the PICU compared to the general floor. As the HFNC guideline gave parameters for titration of HFNC and feeding recommendations, these patients were able to be safely managed and fed while on HFNC in the general inpatient unit.

## Discussion

The results of our observational study support a safe oral feeding regimen for infants with bronchiolitis on HFNC. There was no significant difference in hospital length of stay or days spent NPO. While this seems contradictory to our hypothesized improved care with HFNC, we believe it may be attributed to either our small sample size or that children fed on higher liter flow rates might have stayed even later. Our study was unique in that majority of our patients were managed in the general pediatrics unit. Managing enteral feeding on HFNC provides a unique challenge in that there is limited evidence in general ward feeding practices in pediatric literature and while observing no adverse events, we also found a decreased length of stay in the PICU [5].

The results of our study support both the safety and efficacy of feeding while utilizing HFNC for bronchiolitis. Although we approached these patient populations in both PICU and general pediatrics patients with the oral feeding on HFNC, several papers showed similarly promising results. Sochet et al. [6] investigated 132 infants and found a low incidence of aspiration-related respiratory failure from feeding on HFNC. Their median high flow rate was ~8 L/min, matching our data set and instituted guidelines. In addition, Slain et al. [7], studied a group of 70 infants admitted to the PICU and found a low incidence of adverse events while feeding and shortened PICU length of stay. A study out of Australia prioritized enteral hydration with nasal gastric tube (NGT) and also had zero accounts of aspiration or other adverse events [8]. Although our overall length of stay was not statistically significantly different, using our HFNC guideline showed decreased utilization of PICU.

Our study serves as a validation to the discussion on the safety of oral nutrition in bronchiolitis. Several clinical guidelines waver on the use of early enteral feeding due to the hypothesized amount of positive pressure ventilation delivered by HFNC. Parke et al. [9] observed that for each increase of 10L/min in flow rate, mean airway pressure increased by 0.69cm H2O with subjects breathing with their mouth closed. Nielsen et al. [10] proposed that flow required generating positive end-expiratory pressure (PEEP) of 6 cm H2O was around 5-7 L/min for a term neonate and 14-20 L/min for a toddler. When measuring pressures with the mouth shut, Arora et al [11] showed nasopharyngeal pressures <4.0 cm H2O with flows generated up to 8 L.

Our study has several limitations. First, although our guideline includes flow rates at which to start enteral feeding in infants with bronchiolitis, subjective clinical decision making affected the assessment of a child’s ability to feed at times, whether it be work of breathing, length of illness, or individual comfort. This lack of an objective measure of infantile work of breathing may have limited our ability to initially feed. Although we share a patient population similar to other trials, our study may be inadequately powered to detect the incidence of adverse events in these patients. Existing as a retrospective chart review, this study relied on charting reliability of physicians, nurses, and respiratory therapists. Nevertheless, our data support the limited literature promoting the early, oral feeding of bronchiolitis patients, reporting additional promising statistics in this field.

## Conclusions

Our study provides data to support the safety and efficacy of early oral feeding in infants with bronchiolitis on HFNC. The unique population we observed in the general pediatric unit had strict adherence, based on documentation, to our bronchiolitis feeding guidelines and showed a reduction in PICU utilization. Prospective studies would benefit this field of research by further investigating the effect of pharyngeal pressure and coordination of suck-swallow motions to focus on the risk for aspiration pneumonia, a common fear deterring clinicians from using HFNC. While our study showed the safety of feeding on a range of flow rates, further studies might expand by feeding on higher flow rates during peak illness and following length of stay. HFNC remains a new tool in the arsenal against bronchiolitis, and further studies will continue to justify its success against traditional modes of oxygenation.

## References

[1] Silver AH, Nazif JM (2019). Bronchiolitis. Pediatr Rev.

[2] Ralston SL, Lieberthal AS, Meissner HC (2014). Clinical practice guideline: the diagnosis, management, and prevention of bronchiolitis. Pediatrics.

[3] Jimenez L, Mehta NM, Duggan CP (2017). Timing of the initiation of parenteral nutrition in critically ill children. Curr Opin Clin Nutr Metab Care.

[4] Zaman S, Beardsley E, Crotwell D (2017). Seattle children's bronchiolitis pathway.

[5] Shadman KA, Kelly MM, Edmonson MB (2019). Feeding during high-flow nasal cannula for bronchiolitis: associations with time to discharge. J Hosp Med.

[6] Sochet AA, McGee JA, October TW (2017). Oral nutrition in children with bronchiolitis on high-flow nasal cannula is well tolerated. Hosp Pediatr.

[7] Slain KN, Martinez-Schlurmann N, Shein SL, Stormorken A (2017). Nutrition and high-flow nasal cannula respiratory support in children with bronchiolitis. Hosp Pediatr.

[8] Babl FE, Franklin D, Schlapbach LJ (2020). Enteral hydration in high-flow therapy for infants with bronchiolitis: Secondary analysis of a randomised trial. J Paediatr Child Health.

[9] Parke RL, Eccleston ML, McGuinness SP (2011). The effects of flow on airway pressure during nasal high-flow oxygen therapy. Respir Care.

[10] Nielsen KR, Ellington LE, Gray AJ, Stanberry LI, Smith LS, DiBlasi RM (2018). Effect of high-flow nasal cannula on expiratory pressure and ventilation in infant, pediatric, and adult models. Respir Care.

[11] Arora B, Mahajan P, Zidan MA, Sethuraman U (2012). Nasopharyngeal airway pressures in bronchiolitis patients treated with high-flow nasal cannula oxygen therapy. Pediatr Emerg Care.

